# Anti-Tubercular-Therapy-Induced Severe Thrombocytopenia Presenting as a Vocal Cord Bleed With Massive Hemoptysis Causing Mortality: A Catastrophic Combination

**DOI:** 10.7759/cureus.53752

**Published:** 2024-02-07

**Authors:** Suhail M Shaikh, Saket Toshniwal, Anil Wanjari, Sourya Acharya, Sunil Kumar

**Affiliations:** 1 Medicine, Jawaharlal Nehru Medical College, Datta Meghe Institute of Higher Education and Research, Wardha, IND

**Keywords:** anti-tubercular therapy, drug-induced thrombocytopenia, thrombocytopenia, hemoptysis, hematemesis

## Abstract

Severe thrombocytopenia induced by anti-tubercular therapy (ATT) is a rare but potentially life-threatening complication. Severe thrombocytopenia is a known adverse effect of ATT, but its association with fatal hemoptysis is rare. Hematemesis and hemoptysis are two serious symptoms that indicate bleeding from the upper gastrointestinal and the lower respiratory tract, respectively. We report a rare case of a 65-year-old man, a diagnosed case of tuberculosis on ATT, who presented with massive hemoptysis. On navigating the bleed, the source was found to be a vocal cord bleed, which further led to massive clot formation in the left bronchus, leading to the collapse of the subsequent lung, leading to mortality. This case emphasizes the importance of recognizing ATT as a potential cause of bleeding and considering causes of massive hematemesis that are not gastrointestinal. It also highlights the need for a thorough evaluation of the airway in such patients.

## Introduction

Vocal bleed results from the rupture of a submucosal blood vessel of the vocal cord, usually associated with internal or external laryngeal trauma or impaired coagulation profile [1]. Hematemesis is a potentially life-threatening condition that requires prompt diagnosis and management. The most common causes of hematemesis are peptic ulcer disease, erosive disease, gastritis, Mallory-Weiss syndrome, and esophageal varices due to liver cirrhosis, portal vein thrombosis, and schistosomiasis [2]. The most common causes of hemoptysis are infections, vascular involvement (e.g., pulmonary embolism), malignancy, foreign body aspiration, or trauma. However, hematemesis and hemoptysis can also result from bleeding from non-gastrointestinal sources, such as the oral cavity, nasal cavity (epistaxis), the nasopharynx, the larynx, the trachea, and the bronchi [3,4].

Anti-tubercular therapy (ATT) can have various adverse effects, such as hepatotoxicity, nephrotoxicity, neurotoxicity, and hematological toxicity. Thrombocytopenia and coagulopathy are rare but serious hematological complications of ATT, which can increase the risk of bleeding from various sites [5,6]. These causes are often overlooked or misdiagnosed, leading to delayed or inappropriate treatment. We report a rare case of a 65-year-old man who was taking ATT and presented with massive hemoptysis; he was found to have vocal cord bleed, which further led to the formation of a massive clot extending from the left main bronchus to the left upper lobe bronchus and lower lobe bronchus. To our knowledge, this is an extremely rare case of massive hemoptysis due to vocal cord bleeding leading to catastrophic complications of lung collapse due to massive clot formation followed by mortality secondary to ATT. Such catastrophic complications secondary to ATT have never been reported before.

## Case presentation

A 65-year-old man with a history of hypertension and type II diabetes mellitus presented to the emergency room with multiple episodes of massive hemoptysis. He had a history of evening rise of temperature and cough with expectoration for more than two months, for which he was diagnosed with sputum-positive pulmonary tuberculosis, and the patient was on ATT for the same for two months. The patient was taking tab isoniazid (INH) 300 mg once a day, tab rifampicin (RIF) 600 mg once a day, tab pyrazinamide (PZA) 2000 mg once a day, tab ethambutol (EMB) 1600 mg once a day for two months, and was advised to continue these medicines for six months. He had no history of dysphagia, odynophagia, chest pain, or breathlessness. He had no history of alcohol consumption, smoking, or the use of any analgesic drugs or anticoagulants.

On examination, he was pale, tachycardic, tachypnic, and hypotensive (90/60 mmHg). His oxygen saturation was 93% on room air. His chest auscultation revealed bilateral crepitations and rhonchi. His abdomen was soft and non-tender. He was resuscitated with IV fluids and blood transfusions and started on injection octreotide IV infusion at 50 micrograms per hour as the patient presented with multiple episodes of hematemesis, proton pump inhibitor infusion injection pantoprazole 40 mg IV at 8 mg per hour, antifibrinolytics injection tranexamic acid 500 mg IV thrice a day, injection ethamsylate 250 mg IV thrice a day, antibiotics injection ceftriaxone 1 gm IV twice a day and vitamin K analogue injection menadione 10 mg IV once a day. The patient's laboratory investigations were suggestive of severe thrombocytopenia with deranged coagulation and liver profile; a complement assay was performed and was found to be negative with all other investigations done as shown in Table 1.

**Table 1:** Laboratory parameters of the patient

Investigation	Observed value	Reference range
Hemoglobin(g/dL)	7.4	Male: 13.2 - 16.6, female: 11.6 - 15
Total WBC count (cell/cu.mm )	8,400	4,500 - 11,000
Platelet (per mm3)	8,000	1,50,000 - 3,50,000
Prothrombin time (PT) (seconds)	40.2	9.4 - 12.5
Activated partial thromboplastin time (APTT) (seconds)	44.5	25 - 37
International normalized ratio (INR)	1.57	0.9 - 1.1
C-reactive protein (CRP) (mg/dl)	30.08	<1.0
Erythrocyte sedimentation rate (ESR) (mm/hr)	80	Male: 0 - 15, female: 0 - 20
Serum alkaline phosphatase (U/L)	136	38 - 126
Aspartate aminotransferase (AST) (U/L)	158	Male: 17 - 59, female: 14 - 36
Alanine aminotransferase (ALT) (U/L)	146	Male: <50, female: <35
Serum urea (mg/dl)	22	9 - 20
Serum creatinine (mg/dl)	1.1	Male: 0.66 - 1.25, female: 0.52 - 1.04
Serum sodium (mmol/L)	138	137 - 145
Serum potassium (mEq/L)	3.8	3.5 - 5.1

**Figure 1:**
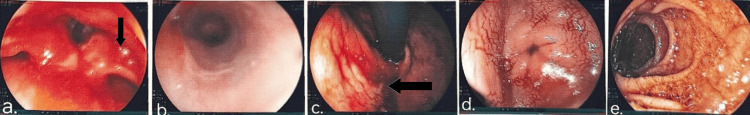
Upper gastrointestinal endoscopy of the patient a) Active bleed probably from the erosive artery near the vocal cord (shown with a black arrow). b-e) Suggestive of fresh pooled blood from the vocal cords in the esophagus and the stomach (shown with a black arrow), with no active bleeding.

The patient’s ATT was withheld in view of the severely deranged liver profile, coagulation profile, and severe thrombocytopenia. The patient underwent an upper gastrointestinal endoscopy, which showed active bleeding around the vocal cords and fresh blood in the esophagus and the stomach. However, no upper gastrointestinal lesions were detected, ruling out a gastrointestinal source of bleeding. The hematemesis was due to swallowed blood from the upper respiratory tract (Figure 1).

**Figure 2:**
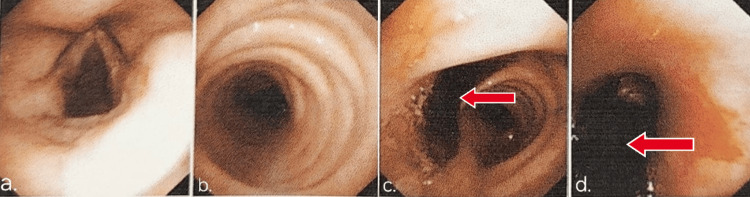
Bronchoscopic images of the patient a) Normal vocal cords with no active bleeding. b) Normal tracheal lumen. c) Massive clot noted in the left main bronchus marked with a red arrow. d) Massive clot extending to the left upper and lower lobe bronchus marked with a red arrow.

The source of the bleeding was the erosive artery near the vocal cord, which was confirmed by a laryngoscopy, and the bleeding was stopped in the same sitting. After a few days of stopping anti-tubercular treatment, the patient’s liver function test came within the normal limit, and his liver enzymes settled within the normal limits. Later, video laryngoscopy was repeated after stabilizing the patient on the sixth day of his admission, which was suggestive of cystine normal vocal cords, and no oozing or bleeding was seen in and around the vocal cord. The patient’s oxygen saturation kept worsening with persistent tachycardia and tachypnea. A bleed in the respiratory tract was suspected, and hence, a bronchoscopy was performed, which revealed a massive clot. The clot was due to accumulating blood in the bronchus and alveoli secondary to the vocal cord bleed. The clot was extending from the left main bronchus to the left upper lobe bronchus and lower lobe bronchus, causing partial obstruction of the airway (Figure 2 and Video 1).

**Video 1:** Bronchoscopy revealing a massive clot in the left main bronchus extending to the left upper and lower lobe bronchus.

A rigid bronchoscopic clot removal from the left main bronchus was planned, but the patient’s condition deteriorated rapidly, and he developed sudden tachycardia, tachypnea, and falling saturation. The patient was intubated and put on a mechanical ventilator in volume control mode. A chest X-ray revealed that the patient’s left lung had collapsed due to the massive clot in it. The patient was started on ionotropic support of injection noradrenaline, vasopressin, and dobutamine continuous infusion due to persistent shock. Despite the best efforts, the patient ultimately succumbed to the complications.

## Discussion

This case illustrates a rare and unusual cause of massive hemoptysis, further leading to bronchial clot formation in a 65-year-old man who was diagnosed and treated as a case of sputum-positive pulmonary tuberculosis and was on anti-tubercular therapy for two months. The patient experienced multiple episodes of hematemesis, which was due to the pooling of blood originating from the vocal cord in the gastrointestinal tract. The etiology of the vocal cord bleeding, in this case, is thrombocytopenia, deranged coagulation, and tuberculosis-related vasculitis, which led to erosion and bleeding from fragile erosive arteries [4]. And, the triggering potential cause of the vocal cord bleed was extensive coughing. Thrombocytopenia, though rare, is seen when a susceptible individual takes the incriminating dose of ATT [7]. After stopping the anti-tubercular drug that was suspected to cause the thrombocytopenia, the platelet counts increased. This confirmed that the anti-tubercular therapy was responsible for the low platelet count in this case [8]. Rifampicin causes thrombocytopenia either due to suppressing platelet production or immunologic destruction of platelets. The complement system activates when rifampicin and antibody molecules bind together and destroy the platelets [9]. ATT contains charged molecules that enable them to bind both to platelet surface proteins and antibodies. Hence ATT binds non-covalently and reversibly to platelets on GP IIb-III or GP Ib-V-IX and also the antibody. This forms the tight bond between the platelet epitope and antibody leading to platelet destruction and thrombocytopenia.

Kuwabara et al. found that isoniazid also causes immune thrombocytopenia by complement activation [10]. The bronchial clot, in this case, was a consequence of the vocal cord bleed, as the blood was continuously aspirated into the bronchus and formed a clot. The diagnosis of vocal cord bleed was made by upper gastrointestinal endoscopy, laryngoscopy, and bronchoscopy, the gold standard methods for evaluating hematemesis and hemoptysis of unknown origin. The differential diagnoses of vocal cord bleed are trauma to the vocal cord, extensive coughing, blunt trauma to the larynx, extensive screaming, upper respiratory tract infection (URTI), the patient being on anti-platelet or anticoagulation medicine, chemical injury due to acid reflux, vocal abuse, and bleeding tendencies such as hemophilia, hemorrhagic purpura, scurvy, and leukemia [4].

The treatment of vocal cord bleed is conservative medical management and bronchial clot is rigid bronchoscopy with cryo-evacuation of the clot. The prognosis of vocal cord bleeding and bronchial clot is generally good as long as the bleeding is controlled and the airway is secured. Unfortunately, in this case, the patient succumbed to the complications secondary to lung collapse due to a huge impacted clot in the left main bronchus extending to the left upper lobe bronchus and left lower lobe bronchus. This case illustrates how ATT can cause severe thrombocytopenia, which can lead to bleeding or bleeding tendencies. There are other side effects of ATT, which need timely monitoring, which include ethambutol-induced optic neuropathy, alteration in liver enzymes, chronic active hepatitis, acute hepatitis, acute kidney injury, aplastic anemia, and peripheral neuropathy [10]. This case highlights the importance of considering different causes of hemoptysis and hematemesis, and of conducting a comprehensive airway assessment in such patients. It also demonstrates how blood from the respiratory tract can pool in the gastrointestinal tract and present like spurious hematemesis. It also underscores the need for a multidisciplinary approach involving gastroenterologists, otolaryngologists, and pulmonologists for the diagnosis and management of massive hemoptysis due to vocal cord bleed and bronchial clots.

## Conclusions

Massive hemoptysis is a critical condition that demands immediate diagnosis and treatment. Hematemesis, besides gastrointestinal etiologies, may result from the accumulation of blood due to hemorrhage from the respiratory tract such as oral cavity, the nasal cavity, the nasopharynx, the larynx, and the bronchi. Also, the causes of hematemesis and hemoptysis can be due to severe thrombocytopenia as a consequence of anti-tubercular therapy. These causes are often overlooked or misdiagnosed, leading to delayed or inappropriate treatment. This case highlights the importance of evaluating the patients on ATT and monitoring them thoroughly to prevent a catastrophic event like in this case that leads to mortality.
